# The ‘shark fin sign’ – a morbid and rare electrocardiographic presentation in takotsubo cardiomyopathy

**DOI:** 10.1093/ehjcr/ytag247

**Published:** 2026-03-30

**Authors:** Mohammad Hamideh, Amna Janjua, Hafez Golzarian

**Affiliations:** Department of Internal Medicine, HCA Houston Healthcare/University of Houston College of Medicine, 22999 US-59 N, Houston, TX 77339, USA; Department of Internal Medicine, HCA Houston Healthcare/University of Houston College of Medicine, 22999 US-59 N, Houston, TX 77339, USA; Department of Cardiovascular Disease, HCA Houston Healthcare/University of Houston College of Medicine, 1313 Hermann Dr, Houston, TX 77004, USA

**Keywords:** Takotsubo cardiomyopathy, Acute coronary syndrome mimic, Electrocardiography

## Vigette

**Figure 1:**
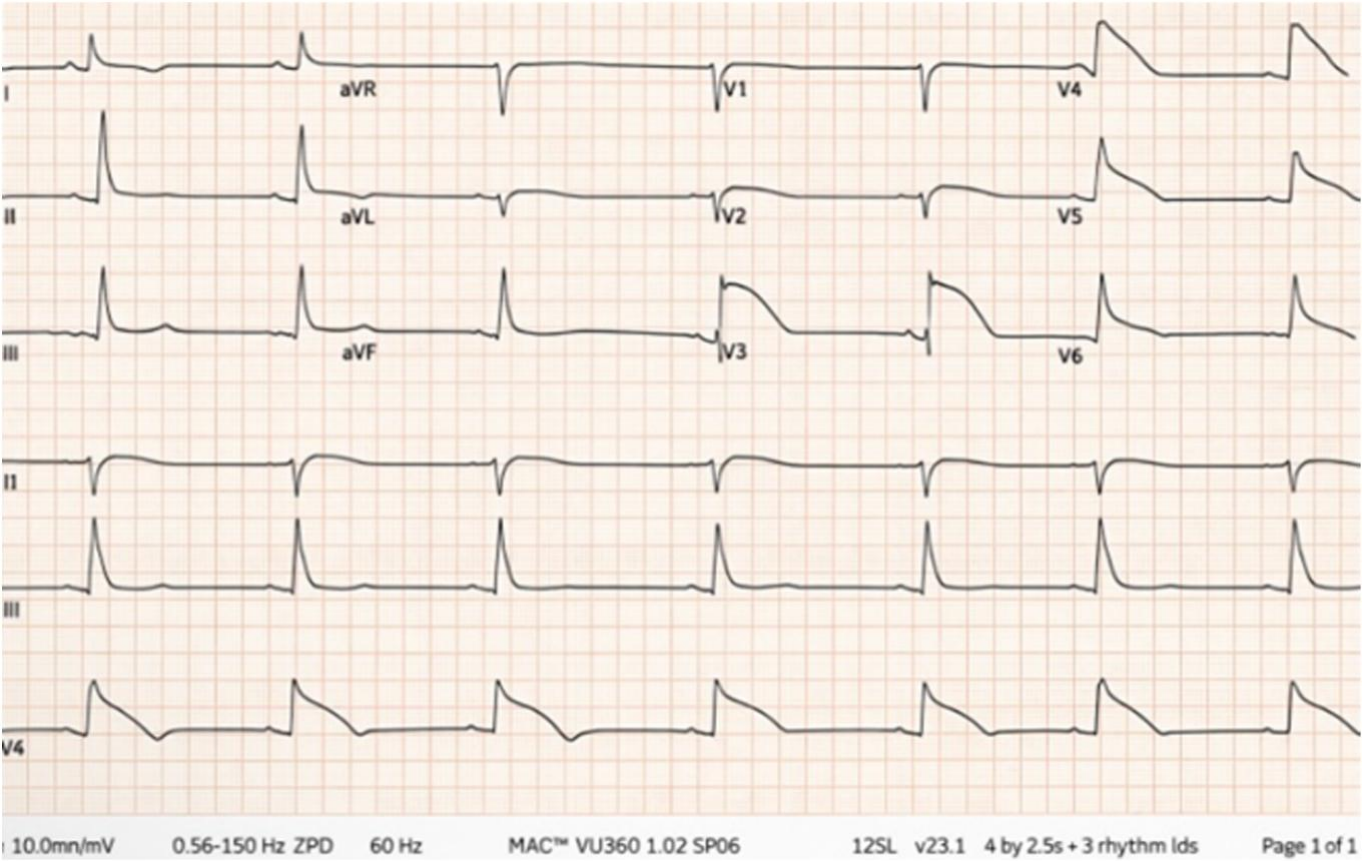
Electrocardiogram revealing marked ST-segment elevation in the anterior leads with morphology mimicking that of a cardiac action potential in lead V3, followed by shark fin morphology in V4-V6.

A middle-aged female with asthma, hypertension, and no prior cardiac history presented to the emergency department with complaints or sudden onset chest pain and shortness of breath. She had been attending to a stressful personal family matter before presentation. Upon arrival, she went into polymorphic ventricular tachycardia (VT) and had cardiac arrest. Return of spontaneous circulation was achieved after eight minutes. Per protocol, an electrocardiogram (ECG) was obtained, with findings as shown in *Figure 1* and Supplementary material. However, recurrent VT persisted, necessitating multiple defibrillations and administration of amiodarone. Laboratory evaluation revealed metabolic acidosis and a mildly elevated high-sensitivity troponin level (84 ng/mL); electrolytes and inflammatory markers were unremarkable. As VT storm persisted, she regressed into cardiogenic shock, necessitating mechanical circulatory support as further diagnostic workup ensued. Emergent coronary angiography confirmed no significant coronary artery disease. Administration of intracoronary nitro-glycerine did not reveal vasospasm. Post-arrest transthoracic echocardiography showed anterior wall and apical hypokinesis with an ejection fraction of 35%. Despite administration of amiodarone and lidocaine, polymorphic VT persisted. Her arrhythmias finally resolved with quinidine administration. History was limited to information provided by her daughter. Home medications included hydrochlorothiazide and as-needed albuterol. Her course was complicated by severe anoxic brain injury, and despite weeks of supportive care, she expired.

## Question 1

In the context of this clinical presentation and ECG, what clinical diagnosis is **most likely**?

- Spontaneous Coronary Artery Dissection (SCAD)
- Prinzmetal Angina
- Brugada Syndrome
- Early Repolarization Syndrome
- Takotsubo Cardiomyopathy

**Correct Answer: (E). Takotsubo Cardiomyopathy**

This ECG represents an extremely unusual and challenging pattern, prompting a meticulous workup and diagnosis of exclusion. Coronary angiography revealed patent coronaries without evidence of dissection or spasm. Furthermore, the absence of ‘tombstoning’ T-waves and reciprocal changes in the inferior leads made options A and B unlikely. Choice C (Brugada Syndrome) is incorrect. Brugada Syndrome does not typically extend to lateral leads, and classically features coved ST segments predominantly in V1-V3, originating from the right ventricular outflow tract—none of which were present here. Choice D (early repolarization) is unlikely as it is generally benign and a normal variant in healthy individuals. Ultimately, such an ECG pattern as above should be considered ischaemic in aetiology until proven otherwise, and emergent coronary angiography should not be delayed.

## Question 2

Which of the following underlying pathophysiologic processes is **most likely** occurring?

- Thrombus formation
- Focal spasm of an epicardial coronary artery
- Sodium channelopathy with increased inward sodium current (I_Na_)
- Tearing of subintimal space in an epicardial coronary artery
- Catecholamine surge

**Correct Answer: (E). Catecholamine surge**

Choice E is correct. The pathophysiology of Takotsubo cardiomyopathy primarily involves a large surge in catecholamines from the adrenal medulla following a stressful event, resulting in myocardial stunning and microvascular spasms. In some cases, a mismatch between endo-epicardial wall tension and stretch-activated channel activation creates a transmural repolarization gradient, resulting in a coved-type ST modification that mimics the shark-fin sign as seen in this case.^1^ This ECG pattern portends a very poor prognosis.^2^ Choice A pertains to occlusive myocardial infarction. Choice B describes Prinzmetal angina. Choice C describes Brudaga. Finally, choice D describes SCAD.

## Question 3

What Vaughan-Williams class of antiarrhythmic drugs constitutes next-line therapy for ventricular arrhythmias refractory to amiodarone?

- Class I
- Class II
- Class III
- Class IV
- Class V

**Correct Answer: (A). Class I.**

Class 1 antiarrhythmics such as lidocaine (1B), procainamide (1A), and quinidine (1A) are sodium channel blockers that effectively treat such ventricular arrhythmias and acute channelopathies by blocking the open sodium channels while simultaneously inhibiting the rapid outward potassium current (It_o_). They are generally recommended as next-line therapy after beta-blockers (Class II) and Amiodarone (Class III).^3,4^ Class IV antiarrhythmics entail calcium channel blockers and are not used to treat ventricular arrhythmias. Class V antiarrhythmics such as adenosine and digoxin are also not indicated in the treatment of ventricular arrhythmias.

## Supplementary Material

ytag247_Supplementary_Data

## Data Availability

The data involved in this case study are available from the authors upon request.

## Notes

Consent: Written informed consent was obtained from the patient(s) for the publication of this case report and any accompanying images or medical information, in accordance with COPE guidelines.
