# Supplementary figures and images for: The ‘shark fin sign’ – a morbid and rare electrocardiographic presentation in takotsubo cardiomyopathy
Source: Eur Heart J Case Rep. 2026 Mar 30;10(4):ytag247. doi: 10.1093/ehjcr/ytag247 (PMC13089528; doi:10.1093/ehjcr/ytag247)

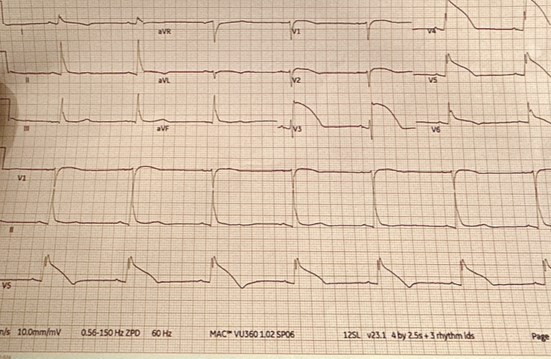

Supplement: ytag247_Supplementary_Data [file ytag247_supplementary_data.jpeg]
